# Coronavirus Disease 2019 and Influenza Vaccination Compliance Among Healthcare Workers at the Primary Health Care Corporation, Qatar, 2020–2024: A Retrospective Study

**DOI:** 10.7759/cureus.85761

**Published:** 2025-06-11

**Authors:** Sameera Alhajri, Anees A Alyafei, Sandy Semaan, Asma A AlNuaimi, Maryam A Al Muslemani

**Affiliations:** 1 Occupational Health and Safety, Primary Health Care Corporation, Doha, QAT; 2 Preventive Medicine/Wellness Programs, Primary Health Care Corporation, Doha, QAT; 3 National Programs Office, Primary Health Care Corporation, Doha, QAT; 4 Planning and Health Intelligence, Primary Health Care Corporation, Doha, QAT

**Keywords:** covid-19 vaccine, healthcare workers (hcws), occupational health, primary health care, seasonal influenza vaccine, vaccination coverage, vaccine uptake

## Abstract

Background

Healthcare workers (HCWs) are a priority population for vaccination due to their high exposure risk and critical role in maintaining healthcare system resilience. While COVID-19 vaccination campaigns have achieved notable success globally, seasonal influenza vaccine uptake remains inconsistent. This study assesses COVID-19 and seasonal influenza vaccination coverage among HCWs at the Primary Health Care Corporation (PHCC) in Qatar between 2020 and 2024, and identifies associated demographic and occupational determinants.

Methods

A retrospective, observational study was conducted using data extracted from PHCC’s electronic medical records system (EMRS) and Human Resources Database (HRD), encompassing 31 health centers. All HCWs employed for ≥3 months were included. Descriptive statistics were used to summarize vaccination rates, and chi-square tests were applied to assess associations between vaccine uptake and variables such as age, gender, nationality, job role, years of service, and educational attainment.

Results

Among 7,463 eligible HCWs (100%), COVID-19 vaccine uptake was high (94.05%), with 18,844 doses administered. Seasonal influenza vaccine coverage was comparatively lower at 65.20%, representing 4,866 HCWs, with 16,533 doses given. Higher age, non-Qatari nationality, female gender (for COVID-19), and shorter service duration were significantly associated with increased uptake (p < 0.05). Educational attainment showed statistically significant associations for both vaccines, although coverage rates were similar across qualification levels. Clinical role was not significantly associated with influenza uptake, but it was significant for COVID-19 vaccination.

Conclusion

COVID-19 vaccination compliance among HCWs at PHCC was robust and aligned with high-income country benchmarks. However, seasonal influenza vaccination remains below the WHO-recommended 75% threshold. Findings underscore the need for sustained institutional efforts to address vaccine fatigue and promote annual influenza immunization, particularly among younger, Qatari, and longer-serving HCWs. Tailored strategies, education, and institutional mandates may enhance future vaccine compliance and protect healthcare system integrity.

## Introduction

Vaccination remains a fundamental pillar of public health, particularly among healthcare workers (HCWs) in primary healthcare settings, who are at increased risk of exposure to infectious diseases. Their frontline role underscores the importance of maintaining high vaccination coverage to prevent nosocomial transmission and ensure the continuity of healthcare services. Seasonal influenza and coronavirus disease 2019 (COVID-19) have posed significant global threats, highlighting the necessity of robust vaccination strategies targeting HCWs [1].

Seasonal influenza vaccination has long been recommended for HCWs as an effective preventive measure, demonstrating benefits in reducing absenteeism, protecting vulnerable patient populations, and maintaining service functionality during peak influenza seasons [2]. Nevertheless, despite national and international guidelines, vaccination rates among HCWs often fall below target levels, influenced by factors such as vaccine hesitancy, concerns regarding adverse effects, and inconsistent institutional mandates [3].

Global vaccination coverage among HCWs remains variable. A meta-analysis by Fan et al. reported an average influenza vaccination rate of 41.7%, with considerable regional differences: the Americas (67.1%), the Middle East (51.3%), Oceania (48.7%), Europe (42.5%), Asia (28.5%), and Africa (6.5%) [4]. Similarly, the rollout of COVID-19 vaccines prioritized HCWs worldwide; however, by 2021, only 33% of HCWs in low-income countries were fully vaccinated, compared to 88% in high-income countries, illustrating stark disparities in vaccine access and acceptance [5].

Multiple factors influence vaccine uptake among HCWs. Zhao and colleagues highlighted that the COVID-19 pandemic initially increased vaccination willingness, yet hesitancy remains an ongoing concern [6]. Demographic factors such as younger age, male gender, and higher educational attainment have been associated with increased vaccine acceptance rates [7,8]. Furthermore, HCWs with advanced degrees tend to express greater trust in vaccine safety and efficacy, whereas knowledge gaps and misconceptions contribute to hesitancy, emphasizing the need for continuous educational interventions [9]. Nationality also appears influential, with higher vaccination rates reported in North America (85.6%) compared to Africa (65.6%) [10,11].

Vaccination of HCWs is associated with decreased patient transmission, fewer nosocomial infections, and reduced mortality, particularly in long-term care facilities [12,13]. In Qatar, the Primary Health Care Corporation (PHCC), the nation’s leading provider of governmental primary care services, has actively promoted COVID-19 and seasonal influenza vaccination campaigns to protect its workforce and patients.

In Qatar, both COVID-19 and seasonal influenza vaccines were provided free of charge by governmental health centers. COVID-19 vaccination was mandatory for all PHCC healthcare workers, both clinical and non-clinical, whereas seasonal influenza vaccination was recommended but not compulsory [1]. In the context of this study, “mandatory vaccination” refers to the institutional policy at PHCC that required at least one documented dose of the COVID-19 vaccine during the designated immunization campaign period.

Initial COVID-19 vaccine uptake was high due to policy mandates and heightened risk perception; however, sustaining booster coverage has presented challenges, including vaccine fatigue and evolving perceptions of disease severity.

Despite these efforts, data on longitudinal vaccination trends among HCWs in Qatar remain limited. Identifying determinants of vaccine uptake is crucial for shaping targeted occupational health policies in the country.

Therefore, this study aims to determine the coverage of COVID-19 and seasonal influenza vaccines among PHCC healthcare workers in Qatar between 2020 and 2024 and to identify the demographic and occupational determinants associated with vaccination uptake.

## Materials and methods

Study design and setting

This is a retrospective cohort study analyzing HCWs' vaccination status at the Primary Health Care Corporation (PHCC) in Qatar. Data were obtained from the electronic medical records system (EMRS) across 31 health centers and the Human Resources Database (HRD). The study analyzed available data from January 2020 to July 2024, encompassing the COVID-19 pandemic period and capturing seasonal influenza vaccination coverage before the COVID-19 crisis.

Study population

The study population included all HCWs employed by PHCC who had been in service for at least three months. Eligible participants comprised clinical professionals, allied health professionals, administrative staff engaged at headquarters or community health centers, and workers involved in direct patient care or exposed to blood and body fluids. Outsourced and temporary staff were excluded. The institutional probation period is three months, after which staff members can be permanently designated as PHCC HCWs. This period allows staff to become familiar with the organization's vaccination campaigns and policies.

COVID-19 vaccination was mandated by national health authorities for all HCWs in Qatar, irrespective of their roles, due to their potential exposure within healthcare environments. In contrast, seasonal influenza vaccination was not mandatory but was recommended for all HCWs, with particular emphasis on those in direct patient care or deemed high risk based on occupational exposure.

Sampling strategy

A universal sampling method was used to include all eligible HCWs during the study period. Since the study involved the entire workforce of PHCC (7,463 individuals), a formal sample size calculation was not required.

Data collection

Data were anonymously collected from two institutional sources: the HRD and the EMRS. Sociodemographic variables included age (in completed years), gender (female or male), and nationality (Qatari or non-Qatari). Career-related variables included job role (clinical or nonclinical). Healthcare workers were defined as all officially employed personnel at PHCC, including clinical staff (physicians, nurses, allied health professionals) and nonclinical staff (administrative, managerial, and support services), years of experience (in completed years), and educational qualifications (below bachelor's degree versus bachelor's degree or higher).

Vaccination records for seasonal influenza and COVID-19 were extracted from the EMRS, including vaccination status (vaccinated or not) and the number of doses administered. All data were securely stored in a password-protected Microsoft Excel® file (Microsoft Corporation, Redmond, Washington), with access restricted to authorized research team members only.

Ethical consideration

The study received ethical approval from the PHCC Institutional Review Board (IRB), ensuring adherence to established research ethics guidelines. All HCW data were anonymized to protect confidentiality.

Quality measures

A standardized data extraction protocol, approved by the IRB, was developed to guide the systematic identification and recording of information from the HRD and EMRS databases. The research team, led by the principal investigator, underwent training to ensure accuracy and consistency in data collection.

Multiple team members conducted regular reliability checks, with discrepancies resolved by consensus or escalated to the principal investigator. Routine audits and validation procedures were performed by cross-referencing extracted data with original EMRS records to confirm accuracy and adherence to the study protocol.

Data analysis

Descriptive statistics were utilized to calculate the characteristics of study participants and vaccination coverage rates in percentages. Continuous variables were presented as means and standard deviations, while categorical variables were presented as frequencies and percentages.

A chi-square test was used to assess the relationship between vaccination status and sociodemographic factors. All statistical analyses were conducted using IBM SPSS Statistics for Windows, Version 29.0.2.0 (Released 2023; IBM Corp., Armonk, New York), with a p-value of <0.05 considered statistically significant.

## Results

Table 1 illustrates a dataset encompassing 7,463 PHCC employees analyzed to evaluate HCWs' demographics. The average age of employees was 40.82 ± 9.00 years. The predominant age group among them was 19-39, accounting for 48.69%, while one-third (33.93%) of the HCWs belonged to the 40-49 age group.

**Table 1:** The Age Groups of Primary Health Care Corporation Workforce, 2024

Age	n (%)	Mean ±SD
19-39	3,634 (48.69)	30.79±5.10
40-49	2,532 (33.93)	44.67±2.37
50-59	1,109 (14.86)	53.90±2.98
≥60	188 (2.52)	62.08±2.10
Total	7,463 (100)	40.82±9.00

Table 2 indicates that the female workforce represents more than two-thirds of employees, with females accounting for the majority of registrations. The nationality analysis showed that most employees were non-Qatari, with 5387 individuals (72.18%). Clinical healthcare workers constituted approximately two-thirds of the PHCC workforce, while nonclinical workers made up 2669 (35.75%). Most nonclinical workers had less than ten years of service. Among the 7463 total employees, nearly three-quarters possessed a bachelor's degree or higher.

**Table 2:** The Characteristics and Vaccination Coverage of Primary Health Care Corporation Workforce, 2024 n: number, HCW: health care workers, PHCC: Primary Health Care Corporation.

Sociodemographics	n (%)
Gender	
Female	4,856 (65.06)
Male	2,607 (34.93)
Nationality	
Qatari	2,076 (27.82)
Non-Qatari	5,387 (72.18)
Job role	
Clinical HCW	4,794 (64.24)
Nonclinical HCW	2,669 (35.75)
Years in PHCC	
0 to 10	5,283 (70.79)
11 to 20	1,541(20.65)
more 21	639 (8.56)
Qualification	
Bachelor’s degree and above	5,562 (74.53)
Less than a bachelor’s degree	1,901 (25.47)
Total	7,463 (100)

Further analysis of vaccination coverage among the 7,463 PHCC staff members showed that seasonal influenza vaccine coverage was 4,866 (65.20%), with a total of 16,533 doses administered. In comparison, COVID-19 vaccination coverage was higher, with 7,019 individuals vaccinated (94.05%) and a total of 18,844 doses administered, indicating that many HCWs received more than one dose. For the purpose of this study, vaccine uptake was defined as the receipt of at least one documented dose of the respective vaccine (COVID-19 or seasonal influenza) during each immunization season.

Table 3 analyzes vaccination uptake for seasonal influenza and COVID-19 across demographic groups using chi-square tests. Older individuals showed higher rates: 77.5% for influenza and over 95% for COVID-19. Males were more likely to receive the influenza vaccine (69.70%), while females had higher COVID-19 vaccination rates (94.73%). Non-Qataris also had higher uptake: 54.31% for influenza and 67.21% for COVID-19. Job role impacted COVID-19 rates, with nonclinical healthcare workers at 54.72% compared to clinical workers at 39.32%. Those with fewer years of service were more likely to be vaccinated. Vaccination rates were around 65% for seasonal influenza and 95% for COVID-19, based on qualifications. All variables except job role in influenza vaccination showed statistically significant differences (p < 0.05), indicating strong associations between these demographic factors and vaccine uptake.

**Table 3:** Association Between Sociodemographic and Vaccination Uptake for Influenza and COVID-19 Among Primary Health Care Corporation Health Care Workers, Qatar, 2024 COVID-19: coronavirus disease 2019, PHCC: Primary Health Care Corporation, HCW: health care worker, df: degree of freedom.

Vaccine	Variable	Category	No. Vaccinated (%)	χ²-Test	df	P-value
Seasonal influenza	Age (completed years)	19-39	2145 (59.02)	564.267	6	<0.001
		40-49	1734 (68.48)			
		50-59	842 (75.92)			
		≥60	145 (77.14)			
	Gender	Female	3049 (62.79)	35.324	1	<0.001
		Male	1817 (69.70)			
	Nationality	Qatari	813 (10.89)	857.290	1	<0.001
		Non-Qatari	4053 (54.31)			
	Job role	Clinical HCW	2475(33.16)	0.172	1	0.6783
		Non-clinical HCW	2391(32.04)			
	Years in PHCC	0-10	3263 (43.72)	553.536	4	<0.001
		11-20	1145 (15.34)			
		≥21	458 (6.14)			
	Qualification	Bachelor’s degree and above	3626(65.19)	732.505	7	<0.001
		Less than a bachelor’s degree	1240(65.23)			
COVID-19	Age (completed years)	19-39	3354(92.29)	55.144	6	<0.001
		40-49	2399(94.74)			
		50-59	1079(97.29)			
		≥60	187 (99.47)			
	Gender	Female	4600 (94.73)	11.073	1	<0.001
		Male	2419 (92.79)			
	Nationality	Qatari	2003 (96.48)	29.861	1	<0.001
		Non-Qatari	5016 (93.11)			
	Job role	Clinical HCW	4449 (92.80)	94.956	1	<0.001
		Non-clinical HCW	2570 (96.29)			
	Years in PHCC	0-10	4868 (65.23)	525.535	4	<0.001
		11-20	1521 (20.38)			
		≥21	630 (8.44)			
	Qualification	Bachelor’s degree and above	5284 (95.0)	1207.839	7	<0.001
		Less than a bachelor’s degree	1824 (96)			

Further analysis was conducted using multivariate logistic regression analysis, which identified several independent predictors associated with an increased likelihood of vaccine uptake among PHCC healthcare workers, as shown in Table 4.

**Table 4:** Comparative Multivariate Logistic Regression Results for COVID-19 and Influenza Vaccine Uptake Among the Health Care Workers in Primary Health Care Corporation HCW: health care worker, COVID-19: coronavirus disease 2019, aOR: adjusted odds ratio, CI: confidence interval.

Predictor Variable	COVID-19			Influenza		
	aOR	95% CI	P-value	aOR	95% CI	P-value
Age ≥50 vs <50	1.87	1.50–2.32	0.001	1.52	1.24–1.86	0.001
Female vs male	1.28	1.09–1.51	0.004	0.93	0.80–1.09	0.391
Non-Qatari vs Qatari	1.64	1.35–1.98	0.0	1.38	1.17–1.62	0.001
Non-clinical HCW vs clinical role HCW	1.31	1.12–1.54	0.002	1.1	0.94–1.29	0.211
Years of service <10 vs ≥10	1.42	1.19–1.71	0.001	1.35	1.14–1.60	0.001
Bachelor's or above vs below	1.05	0.88–1.25	0.532	1.02	0.87–1.20	0.794

Multivariate logistic regression analysis identified several independent predictors of COVID-19 vaccine uptake. Older age (≥50 years) was significantly associated with a higher likelihood of vaccination (aOR = 1.87; 95% CI: 1.50-2.32; p = 0.001). Non-Qatari nationality (aOR = 1.64), female gender (aOR = 1.28), and fewer years of service (<10 years) (aOR = 1.42) were also associated with greater uptake (all p < 0.01). Non-clinical staff had higher odds compared to clinical staff (aOR = 1.31; p = 0.002). Educational level was not a significant predictor (p = 0.532), suggesting comparable uptake across qualification levels.

For seasonal influenza vaccination, age ≥50 remained a strong predictor (aOR = 1.52; 95% CI: 1.24-1.86; p = 0.001), as did non-Qatari nationality (aOR = 1.38; p = 0.001) and fewer years of service (aOR = 1.35; p = 0.001). In contrast to COVID-19 findings, gender, job role, and education level did not demonstrate statistically significant associations with influenza vaccine uptake.

## Discussion

This study assessed the vaccination status of HCWs at the PHCC in Qatar for COVID-19 and seasonal influenza from 2020 to 2024. COVID-19 vaccination coverage was high at 94.05%, aligning with trends in high-income nations where HCW vaccination rates averaged 88% [5]. In contrast, influenza vaccination uptake among PHCC staff was significantly lower at 65.20%, although it exceeds the global average of 41.7% for healthcare professionals [4]. This rate falls short of the World Health Organization's recommended benchmark of 75% for HCWs [14]. Countries with mandatory influenza vaccination policies, like the United States, report uptake rates exceeding 80% [15]. A previous local study found a higher influenza vaccination rate of 77% among HCWs in Qatar for the 2015-2016 season [16]. The decreased uptake observed post-COVID may reflect shifting health priorities, reduced perceptions of influenza severity, vaccine fatigue, and decreased focus on seasonal vaccination.

Vaccine fatigue might be responsible for reducing booster uptake and influenza vaccination rates. Various international research studies have shown this phenomenon, suggesting that ongoing campaigns, changing guidelines, and a perceived decreased risk from emerging COVID-19 variants contributed to declining compliance over time. Implementing targeted educational and motivational strategies, such as reminders and peer modeling, can address this trend and enhance the sustainability of vaccination initiatives within the healthcare workforce [17].

Furthermore, the study aimed to identify the factors associated with primary healthcare workers' vaccination status. The results demonstrated that age and years of experience were positively associated with vaccine uptake for COVID-19 and influenza. Healthcare workers aged 50 and above had significantly higher coverage for both vaccines (COVID-19: 97.73%; influenza: 73.58%), which may be attributed to heightened risk perception and greater health literacy. Research conducted in the United States and China showed similar results, indicating that older HCWs demonstrated greater compliance due to a heightened perception of vulnerability to infection [7]. Research in Saudi Arabia also highlighted age as a crucial factor influencing vaccine uptake, especially concerning seasonal influenza [8].

This study revealed notable gender differences in vaccination uptake, with females showing higher COVID-19 vaccination rates (94.73%) and males having higher influenza vaccination rates (69.70%). These results align with a meta-analysis by Wang et al., which indicated varying vaccine acceptance by gender, influenced by cultural roles and trust in healthcare systems [8]. European data also reflect higher influenza vaccine acceptance among male healthcare workers, possibly due to fewer concerns about side effects [9].

Regarding nationality, non-Qatari healthcare workers exhibited higher acceptance for both vaccines compared to their Qatari counterparts, consistent with global findings that foreign-born healthcare workers often have higher vaccination rates due to institutional mandates. Research in the UAE supports this, showing non-national HCWs adhering more to vaccination protocols than their national peers [6].

The professional role did not significantly correlate with influenza vaccination uptake (p = 0.6783), but nonclinical HCWs had higher COVID-19 vaccination rates than their clinical peers. This contrasts with studies from Germany and Italy, where clinical staff typically had higher rates due to patient contact [1]. These differences highlight the need for tailored strategies to enhance vaccine uptake across all job roles.

This study examines vaccine uptake among 7,463 healthcare workers in Qatar, highlighting trends in COVID-19 and seasonal influenza vaccinations from 2020 to 2024. Its strengths include a representative sample and integration of EMR and HRD data, which aids targeted health interventions.

However, limitations include the retrospective design, which restricts causal inference and may underestimate vaccine coverage by excluding external vaccination events. The study also lacks insights into personal beliefs affecting vaccination decisions.

Future research should adopt prospective methods and include interventions to improve vaccination rates, while subgroup analyses could inform tailored health policies. Integrating behavioral science into vaccine promotion will enhance readiness for future outbreaks.

## Conclusions

In conclusion, this study revealed high compliance with COVID-19 vaccination among HCWs at PHCC, reflecting successful institutional strategies during the pandemic. However, the lower uptake of seasonal influenza vaccines highlights ongoing challenges in sustaining routine immunization efforts in the post-COVID period. Demographic factors such as age, nationality, and gender significantly influenced vaccine acceptance. Addressing vaccine fatigue and enhancing awareness through targeted, evidence-based interventions is essential. Future research should explore behavioral drivers and evaluate tailored strategies to boost influenza vaccine uptake and ensure long-term workforce protection.

## References

[1] Scardina G, Ceccarelli L, Casigliani V (2021). Evaluation of flu vaccination coverage among healthcare workers during a 3 years' study period and attitude towards influenza and potential COVID-19 vaccination in the context of the pandemic. Vaccines (Basel).

[2] Li T, Qi X, Li Q (2021). A systematic review and meta-analysis of seasonal influenza vaccination of health workers. Vaccines (Basel).

[3] Maltezou HC, Poland GA (2016). Vaccination of healthcare workers: a review. Hum Vaccin Immunother.

[4] Fan J, Xu S, Liu Y, Ma X, Cao J, Fan C, Bao S (2023). Influenza vaccination rates among healthcare workers: a systematic review and meta-analysis investigating influencing factors. Front Public Health.

[5] Nabaggala MS, Nair TS, Gacic-Dobo M, Siyam A, Diallo K, Boniol M (2022). The global inequity in COVID-19 vaccination coverage among health and care workers. Int J Equity Health.

[6] Zhao T, Xu Q, Cai X (2025). Global spatio-temporal distribution of coronavirus disease 2019 vaccine hesitancy between 2020 and 2022: A meta-analysis. Vaccine.

[7] Meng L, Bell J, Soe M (2024). Comparison of factors associated with seasonal influenza and COVID-19 booster vaccination coverage among healthcare personnel working at acute care hospitals during 2021-2022 influenza season, National Healthcare Safety Network, United States. Prev Med.

[8] Wang L, Wang Y, Cheng X, Li X, Yang Y, Li J (2022). Acceptance of coronavirus disease 2019 (COVID-19) vaccines among healthcare workers: a meta-analysis. Front Public Health.

[9] Fotiadis K, Dadouli K, Avakian I (2021). Factors associated with healthcare workers’ (HCWs) acceptance of COVID-19 vaccinations and indications of a role model towards population vaccinations from a cross-sectional survey in Greece, May 2021. Int J Environ Res Public Health.

[10] Galanis P, Vraka I, Katsiroumpa A (2022). COVID-19 vaccine uptake among healthcare workers: a systematic review and meta-analysis. Vaccines (Basel).

[11] Schmid P, Rauber D, Betsch C, Lidolt G, Denker ML (2017). Barriers of influenza vaccination intention and behavior - a systematic review of influenza vaccine hesitancy, 2005-2016. PLoS One.

[12] Ahmed F, Lindley MC, Allred N, Weinbaum CM, Grohskopf L (2014). Effect of influenza vaccination of healthcare personnel on morbidity and mortality among patients: systematic review and grading of evidence. Clin Infect Dis.

[13] Fox AM, Choi Y, Lin L (2023). Substantial disparities in COVID-19 vaccine uptake and unmet immunization demand in low- and middle-income countries. Health Aff (Millwood).

[14] Feng LZ, Jiang HY, Yi J (2022). Introduction and implications of WHO position paper: vaccines against influenza, May 2022 (Article in Chinese). Zhonghua Yi Xue Za Zhi.

[15] Bell J, Meng L, Barbre K (2023). Influenza and up-to-date COVID-19 vaccination coverage among health care personnel - National Healthcare Safety Network, United States, 2022-23 influenza season. MMWR Morb Mortal Wkly Rep.

[16] Elawad KH, Farag EA, Abuelgasim DA (2017). Improving influenza vaccination rate among primary healthcare workers in Qatar. Vaccines (Basel).

[17] Khubchandani J, Sharma S, Price JH, Wiblishauser MJ, Sharma M, Webb FJ (2021). COVID-19 vaccination hesitancy in the United States: a rapid national assessment. J Community Health.

